# Supplementary material for: Stacking Model‐Based Classifiers for Dealing With Multiple Sets of Noisy Labels
Source: Biom J. 2025 Mar 12;67(2):e70042. doi: 10.1002/bimj.70042 (PMC11898607; doi:10.1002/bimj.70042)
Supplement: Supplementary file 1 — Supporting Information [file BIMJ-67-e70042-s001.zip › density-based-ensemble-model-main/competitors_real_data/netlab-master/netlab-master/help/demnlab.htm]

Netlab Reference Manual demnlab


# demnlab

## Purpose

A front-end Graphical User Interface to the demos

## Synopsis

```
demnlab
```

## Description

This function will start a user interface allowing the user to select
different demonstration functions to view. The demos are divided into 4
groups, with the demo being executed by selecting the desired option
from a pop-up menu.

## See Also

---

**Pages:**
Index


---

Copyright (c) Ian T Nabney (1996-9)
